# Framing the process in the implementation of care for people with generalized anxiety disorder in primary care: a qualitative evidence synthesis

**DOI:** 10.1186/s12875-020-01307-6

**Published:** 2020-11-20

**Authors:** Ana Toledo-Chávarri, Vanesa Ramos-García, Alezandra Torres-Castaño, María M Trujillo-Martín, Wenceslao Peñate Castro, Isabel Del Cura-Castro, Pedro Serrano-Aguilar, Lilisbeth Perestelo-Pérez

**Affiliations:** 1 Canary Islands Health Research Institute Foundation, Tenerife, Spain; 2 Research Network on Health Services in Chronic Diseases (REDISSEC), Madrid, Spain; 3 Center for Biomedical Research of the Canary Islands (CIBICAN), Tenerife, Spain; 4 The Spanish Network of Agencies for Health Technology Assessment and Services of the National Health System (RedETS), Tenerife, Spain; 5 Facultad de Ciencias de la Salud – Sección de Psicología, University of La Laguna, Tenerife, Spain; 6 Unidad de Apoyo a la Investigación, Gerencia Asistencial de Atención Primaria, Madrid, Spain; 7 Department Preventive Medicine and Public Health, University Rey Juan Carlos, Madrid, Spain; 8 Evaluation Unit (SESCS), Canary Islands Health Service (SCS), Tenerife, Spain

**Keywords:** Generalized Anxiety Disorder, Experiences and Patient Preferences, Primary Care, Qualitative Evidence Synthesis

## Abstract

**Background:**

Generalized anxiety disorder (GAD) is one of the most common mental disorders in primary care (PC). GAD has low remission and high relapse rates over long follow-up periods. Qualitative evidence was synthesized to understand the implementation of care and treatment options for people with GAD in PC.

**Methods:**

Research published from 2008 to September 2020 was searched in five databases (MEDLINE, EMBASE, CINAHL, WOS and PsycArticles). Studies that used qualitative methods for data collection and analysis to investigate the implementation of care and treatment options for people with GAD in PC and outpatient settings were included. Non-qualitative studies, mixed methods studies that did not separately report qualitative findings and studies in languages other than English or Spanish were excluded. We used the Confidence in the Evidence from Reviews of Qualitative Research (CERQual) framework to assess the overall confidence in the findings.

**Results:**

The results with a moderate level of confidence showed that the trajectory of care for people with GAD in PC and outpatient settings is long and fluctuates over time, involving multiple difficulties in accessing and maintaining initial treatment or successive treatment options. In addition, there are wide variations in the preferences for and acceptability of different treatment options. The results with a high level of confidence indicated that more information on GAD and its treatment options is needed for PC practitioners, GAD patients and their carers. The results with a low level of confidence suggested that patients use antidepressants for longer than recommended and that the interruption of treatment is not usually planned.

**Conclusions:**

Initial resistance to new treatments among people with GAD can make access and adherence to treatment difficult. Improving care may require patients to be informed of possible trajectories in stepped care pathways before the initiation of treatment so they are aware that they may need to try a number of options until the most effective treatment for them is found. Increased awareness of and information materials on GAD may facilitate both appropriate diagnosis and long-term care.

**Supplementary Information:**

The online version contains supplementary material available at 10.1186/s12875-020-01307-6.

## Background

Epidemiological studies show that anxiety disorders (ADs) are the most common type of mental disorders in the European Union, with a 12-month prevalence of approximately 14% and 61.5 million persons affected [1], and are associated with a high burden of illness [1–3]. According to the data provided by the World Health Organization (WHO), more than 260 million people in the world currently live with AD [4]. Generalized anxiety disorder (GAD) is one of the most common ADs [5, 6] and affects women twice as much as men [7]. European epidemiological studies have found a 12-month prevalence of GAD of 1.7 to 3.4% [1] and a lifetime prevalence of 4.3 to 5.9% [8].

GAD is characterized by a persistent, excessive and exaggerated worry about everyday life events for no apparent reason [9]. Excessive worry is typically accompanied by an “on edge” feeling, muscular tension, chronic fatigue, and irritability that causes distress for at least 6 months, which impairs activities of daily living and decreases the quality of life [5, 9]. Patients with GAD often live in a hyperalert state, scanning the environment for possible threats, whether real or perceived [7]. Patients with GAD have difficulty managing uncertainty given that they will often presume danger or threats without evidence of such [9]. Frequently, GAD is associated with other anxiety disorders (e.g., posttraumatic stress disorder or obsessive-compulsive disorder) and mood disorders [7, 10, 11] and shows a high correlation with depressive disorders [1, 12].

GAD is one of the most common mental disorders observed in primary care (PC), with approximately 5% of people receiving PC services having a GAD diagnosis [12], and uses a significant amount of health resources despite the low recognition rate of the disorder [13]. People with GAD visit PC for physical somatic symptoms related to their anxiety disorders, such as headaches or backaches, gastrointestinal (e.g., dyspepsia), cardiovascular (e.g., palpitations) or respiratory (e.g., dyspnea) problems [14, 15]. Misdiagnosis of GAD in PC hinders the choice of appropriate treatment and health improvement [8, 16].

Available treatments for GAD include pharmacological and psychological interventions, either in isolation or in combination [17]. Selective serotonin reuptake inhibitors (SSRIs) are generally considered first-line treatments for GAD [17, 18]. Serotonin–noradrenaline reuptake inhibitors (SNRIs) and pregabalin are also considered first- or second-line treatments. Cognitive behavioural therapy (CBT) is the psychological first-line treatment for GAD. Self-help, psychoeducational groups and applied relaxation are also recommended [18]. Acceptance-based behavioural therapy, meta-cognitive therapy, and adjunctive mindfulness-based cognitive therapy have also shown efficacy in treating GAD [17]. Recent guidelines also recommend a stepped-care model were primary care may have a stronger role in the care pathway of people with GAD. The 2 first steps of the care for GAD include, in the first place, the identification, assessment, active monitoring and referral for additional assessment and intervention, and, secondly, self-help that may be individual non-facilitated or facilitated, psychoeducational groups, self-help groups [19].

Qualitative findings can help to understand the effectiveness of interventions by providing information about the broader context that interventions are associated with and how individual characteristics may influence attitudes towards them. Qualitative evidence synthesis (QES) can shed light on how interventions can be implemented and how, for whom and in what contexts they work [20]. The QES presented here was conducted to inform the formulation of recommendations using the GRADE *Evidence To Decision framework* [21] to be included in clinical practice guidelines (CPGs) for the treatment of GAD in PC. The principal objective of our QES was to locate, appraise and synthesize qualitative evidence to understand the implementation of care and treatment options for people with GAD in PC and outpatient settings.

## Methods

A QES was conducted following the guidance of the Cochrane Qualitative and Implementation Methods Group [22].

### Design

The research question was defined using the SPICE format [23]: *Setting*, PC; *Perspective*, adults (> 18 years old) with GAD, their family members and caregivers, and healthcare professionals attending patients with GAD; *Phenomenon of Interest*, the implementation of care and treatment options; *Comparison*, inpatient mental health settings; *Evaluation*, experience and trajectory of care, acceptability, feasibility and impact on the equity of treatment options [22].

### Data sources and search strategy

Initially, we performed an exploratory scoping review in PubMed; Google Scholar; and other sources of grey literature, such as the Departments of Health of the Autonomous Communities of Spain. Second, we searched the following databases for publications from 2008 to September 2020: MEDLINE (Ovid SP), EMBASE (Elsevier), CINAHL (EBSCOhost), WOS (Clarivate Analytics) and PsyArticles/PsyInfo (EBSCOhost). Search strategies were developed for each database in collaboration with a librarian and included controlled vocabulary (MeSH terms) together with free text terms around the following keywords: generalized anxiety disorder, primary health care and qualitative methodology. The search was limited to publications in English and Spanish. The search strategy used for EMBASE is available in Additional file 1. Reference lists of included papers were also searched for additional studies.

### Selection criteria

Studies were included if they used qualitative methods for data collection (e.g., open, structured or semi-structured interviews; focus group discussions; diaries; document analysis, open-ended survey questions; and observation) and data analysis (e.g., thematic analysis, framework analysis or grounded theory) and if they were conducted to inform the implementation of care and treatment options for people with GAD in PC. The study selection criteria proposed by the Cochrane Qualitative and Implementation Methods Group were used [20]. Due to the scarce available literature, to find an adequate size pool of relevant data, some studies with indirect relevance were included when they provided information for the implementation of care and treatment options for people with GAD in PC. Specifically, studies that included patients with AD or mental health problems or that were conducted in outpatient settings (community health and/or mental health units outside inpatient mental hospital settings) were included. Non-qualitative studies, mixed methods studies that did not separately report qualitative findings and studies in languages other than English or Spanish were excluded.

### Study selection process

In the first phase, two reviewers independently screened the titles and abstracts of all references retrieved via the search strategy. Second, the same two reviewers independently assessed the full-text articles selected in the first step for inclusion. The final selection was made through discussion with the research team until consensus was reached based on the study eligibility criteria.

### Critical appraisal

Two reviewers independently assessed the methodological quality of each included study using the Spanish version of the Critical Appraisal Skills Programme (CASPe) tool for qualitative studies [24]. Disagreements between reviewers were discussed by a third reviewer until consensus was reached. No papers were excluded from the review on the basis of quality.

### Data extraction and synthesis

The QES was performed using a three-stage thematic synthesis process [23]. In the first stage, codes were inductively derived from the data through an iterative process of attributing codes to small sections of meaning within the text, moving back and forth among the studies and constantly comparing the assigned data and codes. Two reviewers independently coded each study using NVivo 12 software. The codebook was shared among the wider group of authors and agreed upon. Second, codes were grouped into logical and meaningful groups in a hierarchical tree structure to form descriptive themes and sub-themes. Finally, analytical themes were developed and examined by all authors until the themes were finalized. Only findings relevant to the treatment of GAD in primary care were synthesized.

We used the Confidence in the Evidence from Reviews of Qualitative Research (CERQual) framework to conduct a final assessment of the overall confidence of the evidence [22, 25]. The identified themes were presented in tables with descriptions of the following characteristics: 1) Summary of the findings, 2) Studies contributing to the review findings, 3) Methodological limitations, 4) Relevance, 5) Coherence, 6) Adequacy, and 7) Evaluation of confidence in each finding. The confidence in each finding was rated as high, moderate, low, or very low. The evaluation of relevance took into account whether the findings were totally or partially indirectly related to GAD based on the study populations and settings.

## Results

### Summary of the selected studies

**Fig. 1:**
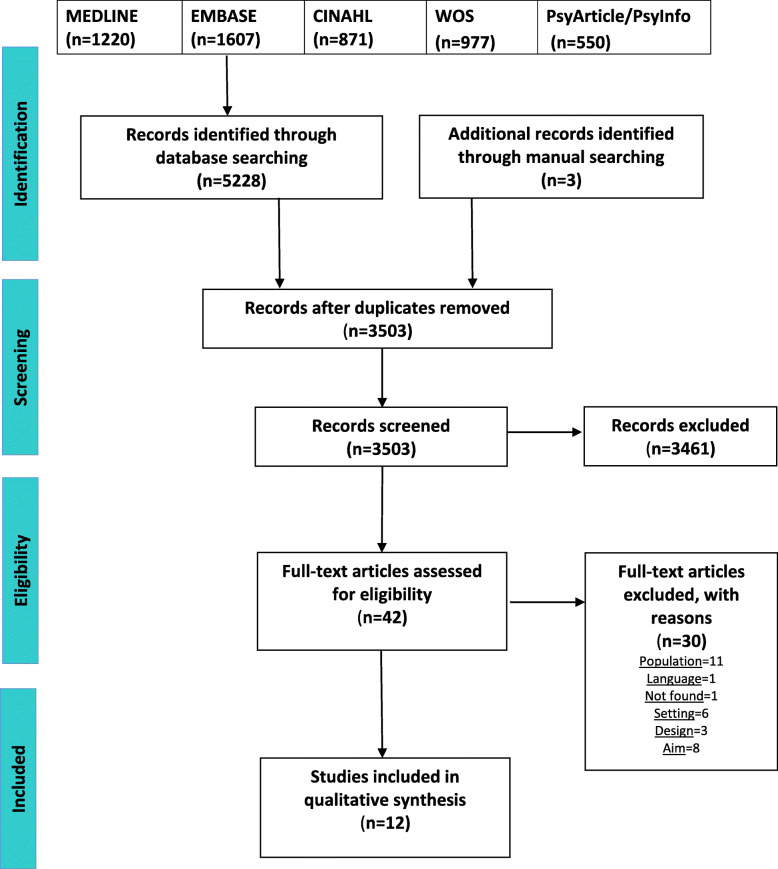
Flow diagram of the selection process of studies

After 3503 records were screened and the references of the included studies were checked, 12 full-text articles were included in the review (see Fig. 1 PRISMA diagram). Three of these 12 studies were additional studies identified and included after manually expanding the literature search. Table 1 shows the main characteristics of the 12 included studies.

**Table 1:** Characteristics of the studies

Author, year and country	Aim of the study	Qualitative study design	Sample (***n***)	Setting	Methodological limitations (CASPe)
Amor 2018 Spain	Include the point of view of a group of patients with GAD in the preparation of the recommendations of a clinical practice guideline.	Focus groups and content analysis. The testimonies of users were linked to recommendations based on the evidence, with both sources of information placed at the same level of relevance.	Patients with GAD (10).	Community mental health unit, Malaga-Center, University Regional Hospital of Malaga.	The design and use of qualitative methodology is adequate, but the objectives are not clearly defined; the methodological objectives are mixed with the research objectives. The presentation of main findings could have been more detailed.
Berg 2010 Sweden	Understand how people experience body psychotherapy focused on affect for one year and to investigate whether treatment improves people’s ability to redefine their somatic symptoms in a broader psychosocial context.	Semi-structured interviews.	Patients with GAD (61).	Psychiatric service, intervention performed by physiotherapists.	The design and use of qualitative methodology is adequate. There are no ethical considerations or reflexive analyses.
Bosman 2016 Holland	Understand the motivations of people and GPs for the long-term use of antidepressants and possible ways to prevent unnecessary use.	Semi-structured interviews in depth and constant comparative analysis.	Patients with GAD and/or depression and > 6 months of antidepressant use (30). GPs (20)	PC network of the Amsterdam Medical Center of the University of VU and University Medical Center of Groningen.	The design and use of qualitative methodology is adequate. There are no ethical considerations or reflexive analyses.
Button 2019 Canada (Toronto)	Examine the experience of patient with GAD about Cognitive Behaviour Therapy (CBT) or Motivational Interviewing (MI).	Semi-structured interviews.	Patient with GAD (10)	Different places in Toronto.	The design and use of qualitative methodology is adequate. Neither ethical considerations nor reflexive analyses are presented
Cook 2007 USA (Philadelphia)	Understand the factors that influence the chronic use of benzodiazepines in older adults.	Semi-structured interviews.	GPs (33)	PC.	The design and use of qualitative methodology seems to be adequate and to meet the minimum aspects of methodological quality. There are no ethical considerations or reflexive analyses.
Cramer 2014 United Kingdom	Examine the experiences and perceptions of men regarding the support they receive in groups and understand the role of professionals in access to these services.	Interviews and observations.	Patients with GAD or depression (17)*	Community services of the town hall.	The design and use of qualitative methodology is adequate. The presentation of the results obtained is also adequate. A section on ethical issues is included, but reflexivity is not assessed.
Dickinson 2010 United Kingdom	Explore the attitudes of older people and their general practitioners regarding the long-term use of antidepressant drugs and their experiences with the influences of the long-term use of these drugs.	Semi-structured interviews.	Patients with AD and depression aged ≥75 years (36) GPs (10)	PC.	The design and use of qualitative methodology is adequate. The findings are clearly stated. Neither ethical considerations nor reflexive analyses are presented.
CPG GAD elaboration group 2008 Spain	Collect as much information as possible about the experiences of people with the disease and their relationship with the health system.	Group discussion with patients and in-depth interviews with PC and specialist health professionals.	Patients with AD, GPs, psychiatrists and psychologists (not reported)	Health areas of the Community of Madrid.	The design and use of qualitative methodology is adequate, but the objectives and analysis are not clearly defined. The findings are clearly stated. Neither ethical considerations nor reflexive analyses are presented.
Health Quality Ontario 2017 Canada (Ontario)	Explore the underlying values, needs, impacts and preferences of those who have experience with major depressive disorder and GAD and the psychological treatments for these disorders. The focus of treatment was CBT, interpersonal therapy and supportive therapy.	Semi-structured interviews.	Patients with GAD and/or depression (20)	Different places in Canada.	The design and use of qualitative methodology is adequate. Neither ethical considerations nor reflexive analyses are presented.
Hurtado 2020 Spain	Understand the experience and perceptions of people diagnosed with GAD, along their trajectory through health services and their role through the decision-making process for their treatment.	Focus group.	Patient with GAD (24)	Service of Mental Health of the Regional University Hospital of Malaga and in the Health Care Area Norte de Córdoba.	The design and use of qualitative methodology is adequate. Neither ethical considerations nor reflexive analyses are presented.
Marcus 2011 Canada	Identify how people perceive the motivational interview process before CBT.	Semi-structured interviews.	Patients with GAD (8)	Private psychological service.	The design and use of qualitative methodology is adequate; no answers are given to the questions that were raised at the beginning of the study. It is not explicit if a coding strategy in pairs was used to minimize the bias of the researchers. Neither ethical considerations nor reflexive analyses are presented.
O’Brien 2017 Ireland	Describe the possible difficulties that a group of participants may have in the context of emotion-focused therapy.	Observations based on multiple case studies.	Patients with GAD (14)	PC psychology services.	The use of qualitative methodology is adequate, except in the design of the data collection. Therapeutic sessions are analysed, but there is no description of how they are carried out. A possible source of bias is that the analysis is conducted based on a theoretical framework previously constructed by one of the authors who trained the others in its application in the review of the data.

* CBT: Cognitive Behavioural Therapy; CPG GAD: Clinical Practice Guidelines for the Management of Patients with GAD in Primary Care; GAD: Generalized Anxiety Disorder; AD: Anxiety Disorder, GP: General Practitioner; PC: Primary Care; MI: Motivational Interviewing; n = sample size

### Country

Of the 12 included studies, 3 were carried out in Spain [26–28], 2 were carried out in the UK [29, 30], 3 were carried out in Canada [31–33], 1 was carried out in Sweden [34], 1 was carried out in the Netherlands [35], 1 as carried out in Ireland [36] and 1 was carried out in the United States [37].

### Objective

Seven studies [26, 27, 31–34, 36] reported patients’ experiences with GAD and their attitudes towards medication and psychotherapy. Three others [29, 30, 35] included both patients’ and professionals’ perspectives and their attitudes towards medication and psychotherapy, and one study described professionals’ and patients’ experiences with GAD and the healthcare system [28]. The last study [37] included only the perspective of physicians regarding the chronic use of medication among elderly people.

### Data collection technique

Most of the included studies used interviews, mainly semi-structured interview [29–35, 37]; 2 studies employed focus groups [26, 27], and one study was based on a case study [36].

### Setting

Four studies were conducted in PC [30, 35–37], four studies were conducted in outpatient mental health services [26–28, 34], one study was conducted in municipal community services [29], one study was conducted in metropolitan outpatient psychiatric services [32], and two studies was carried out in a non-specified outpatient setting [31, 33].

### Population

Six studies included only patients with GAD [26, 27, 31, 32, 34, 36], while another study included general practitioners (GPs) [37]. The rest of the studies included a combined sample of patients with GAD, anxiety disorders or depression and GPs [29, 30, 33, 35]. One study explored only male patients, one study [29] also included psychiatrists and psychologists [28], and two studies included exclusively older people’s perspectives [30, 37].

### Integrated themes

Based on the synthesis of the qualitative evidence, the findings were organized under the following themes: (1) Trajectory of care, (2) Information needs, (3) Acceptability of, preferences for and implementation considerations for treatment options; and (4) Interruption of antidepressant treatment. The findings categorized under each theme and the confidence assessment for each finding are shown in Tables 2–5.

### Theme 1: trajectory of care

People with GAD might experience multiple episodes of the disorder over their lifetimes when facing long care trajectories [28, 35]. The experience of constantly looking for treatments was common. Finding an accessible, adequate and effective treatment could make people with GAD become involved in a long process of searching for, accessing and changing different therapeutic options [26, 27, 33].

The start of any new treatment tended to cause resistance and concern in patients with GAD due to the uncertainty. The degree of concern depended on previous knowledge of and the satisfaction or unsatisfaction previous experiences with different treatments. However, this initial concern tended to disappear over time [26, 28, 33, 34, 36].

### Theme 2: information needs

People with GAD expressed the need to receive more information about the disorder they suffered, the places where they could get help and the kind of help that was available [27, 30, 33]. Additionally, a lack of information on treatment effectiveness was identified as a barrier [33].

Family members and caregivers who supported people with GAD were also interested in receiving information about the disorder, its management and what to do during a crisis. However, people with GAD did not always have or want this support, as the social or family context could sometimes be a source of concern [27–29, 35]. GPs also had information needs; specifically, they expressed doubts about how to act during anxiety crises and relapses [28, 35, 37].

### Theme 3: preferences for, acceptability of and implementation considerations for treatment options

There was individual variability regarding treatment preferences. Some people with GAD preferred pharmacotherapy as a main treatment or as a complement to psychotherapy. However, others showed greater preferences for psychological treatments [26–28, 33]. Receiving the preferred treatment increased the perception of efficacy. Preferences and acceptability affected adherence to pharmacological or psychological treatments [27–29].

Five studies [29, 30, 33, 35, 36] examined experiences related to pharmacological treatment. Bosman et al. [35], Dickinson et al. [30], and Cook et al. [37] pointed out that the acceptability of a drug depended on the perceptions of both the patient and GP regarding its effectiveness for symptom relief. The acceptability of a drug was reduced when people with GAD perceived that it had a limited long-term capacity to eliminate their health problems [27]. Drugs were well accepted if GAD was thought to stem primarily from a physical or medical cause rather than a psychological cause [30].

Some people expressed few or no concerns when starting pharmacological treatment, while others rejected drugs and refused to take them, as they were worried about their side effects and the stigma associated with their use [32, 33, 35, 37].

GPs tended to recognize that social and emotional problems required nonpharmacological interventions that they could not always provide and expressed discomfort when prescribing a drug in those situations [30].

People with GAD reported both positive and negative experiences with psychological therapies [31, 33, 34]. The acceptability of psychological therapies depended on people’s previous experiences [33, 34]. Nevertheless, some people with GAD expressed a strong preference for psychological treatment [27]. Empathy, trust and honesty facilitated acceptance and adherence to these therapies [31, 32, 34, 35]. Characteristics such as distrust, pessimism or directive and judgmental stile were highlighted as negative experiences [31, 33], while involve mutual efforts and engagement by both therapist and patient were positive experiences [31].

In addition, contextual characteristics such as comfort with the environment and therapeutic space were well evaluated by patients with GAD and influenced the acceptability of psychotherapy [32]. The most accepted relationships with health care professionals were with clinical psychologists, especially if they had specialized training and therapeutic experience [32, 33]. One study analysed an intervention conducted by physiotherapists, with whom patients’ relationships seemed to be more variable [34].

People with GAD differed in the type of preferred psychotherapy. CBT, interpersonal therapy and group support or therapy were perceived to be more effective than other types of psychotherapy [27, 28, 33]. Only one study analysed experience with CBT and Motivational interview (MI) [31], though none of the included studies analysed experiences interpersonal therapy. Group support and group therapies were valued as positive experiences, as they allowed the development of social support networks and helped people overcome social isolation [27, 29].

Motivational interviews (MIs) were found to be an acceptable form of therapy [32]. The application of four MIs sessions before CBT increased awareness, impulsiveness, motivation, and confidence for the CBT psychological intervention. Characteristics such as an increase in self-awareness about a problem, a reduction in symptoms of anxiety and worry, and more concrete thoughts about how to manage their conditions were aspects highlighted by the interviewees with GAD. When MIs were performed, a positive relationship with the therapist was a relevant success factor for the therapy [32]. The integration of CBT-MIs, allowed people with GAD to assume a more active and engaged role in treatment. Tools and strategies to cope with worry (i.e., thought record, relaxation, behavioural experiments) were necessary conditions for acceptability of MI-CBT or CBT [31].

Emotion-focused therapy (EFT) was evaluated in one study [36]. The acceptability of EFT depended on the patient’s interpersonal difficulty expressing emotions [36]. GAD patients’ experiences and reported acceptability of affect-focused body psychotherapy (ABP) was variable [36]. The acceptability of ABP was related to the previous experiences that people had with their own bodies and their ability to open up to new therapies [34]. Some people with GAD reported that the therapy increased their stress levels, while other patients reported difficulties finding the connection between concrete situations in their lives and feeling their bodies become tense [36]. However, other GAD patients managed to integrate the perceptions of their own body with their experiences and emotions, allowing them to reconceptualize their bodily sensations as signs of meaning and not as generators of anxiety [36].

### Theme 4: interruption of antidepressant treatment

None of the included studies analysed barriers to adherence to or voluntary interruption of antidepressant treatment.

The interruption of antidepressant treatment is not usually systematized in PC. Practices related to treatment interruption were very variable depending on the GP and his or her relationship with the person with anxiety [30, 33, 37]. Medication was either passively or actively maintained by the GP. Factors affecting the passive maintenance of medication were inertia, routinization and forgetfulness [32, 37]. Active medication maintenance was based on an assessment of the risk/benefit balance performed by GPs and patients that reflected the value of continuing with the medication in view of the perceived benefits [30, 35, 37].

Perception of functional improvements such as a reduction in anxiety symptoms, improved sleep or increased stability were the basis of doctors’ and patients’ preferences to maintain antidepressant treatment [30, 35, 37]. Likewise, possible dependence on or abuse of these drugs was a factor that was viewed negatively [30, 35]. In some cases, antidepressants were the only available treatment, and the GP refused to interrupt the patient’s use of them due to a lack of access to other therapeutic alternatives [30].

One of the factors that hindered the interruption of pharmacological treatment was relapse. Relapse after attempting to stop treatment reinforced patients’ and GPs’ decisions to continue the use of medication [30, 35, 37]. However, one article pointed out that relapses can be a consequence of continuous, uninterrupted treatment [30]. Older patients could have additional barriers to interrupting pharmacological treatment, such as pessimism due to the chronicity of the disease [30]. However, GPs perceived that people of advanced age had a lower risk of drug addiction [30].

When planning to interrupt medication, both GPs and GAD patients tried to identify a suitable time in terms of the patient’s stability and an appropriate reason to start the interruption of treatment [35]. An automated reminder could help GPs start the process of interrupting medication. An appointment specifically scheduled to review the long-term use of antidepressants allowed renegotiation of the treatment plan, including interruption [35].

Both GPs and patients reported needing information about the recommended duration of antidepressant treatments and reasons that should guide their discontinuation. In addition, they also needed information about treatment effectiveness and potential associated side effects in the short and long term. Specifically, information about the possibility and symptoms of dependence as well as about the expected effects associated with medication withdrawal was needed [30, 35, 37].

## Discussion

The findings indicate that the trajectory of care for people with GAD is long and fluctuates over time, involving multiple difficulties in accessing and maintaining initial treatment or successive treatment options. The literature also reports low remission and high relapse rates over long follow-up periods [38]. The remission rate was reported to be approximately 0.38% after five years of prospective follow-up [39]. More than 74% of GAD patients continued to have symptoms after tracking them for 12 years [40]. Additionally, 25% of GAD patients who stopped treatment relapsed within 1 month, and up 80% relapsed within 1 year [41]. More information on the disorder and its treatment options is needed both for GP and GAD patients and their carers. Better awareness and information materials on GAD may facilitate both early appropriate diagnosis and long-term care. The early detection of AD in PC should be improved, as a recent study shows that only 25% of cases are correctly identified in the PC setting [42].

The *Evidence to Decision framework* includes the analysis of acceptability, feasibility and impact on the equity of treatment options [43]. Acceptability aspects can be broadly informed by this review. The findings of our review show that there is wide variability in preferences for and the acceptability of different treatment options. The variable acceptability of treatment options can be a barrier to feasibility and continuity of care, as it affects uptake and adherence to treatment and probably attendance of PC. Difficulty accepting new treatments among people with GAD can hinder the trajectory of care and may require patients to be informed about stepped care pathways before the initiation of treatment [44] to allow them to be prepared to try a number of treatment options until the most effective treatment for them is found. Acceptability also varies over time, which is probably related to high treatment dropout, especially in pharmacological treatments. For example, the dropout rate of SSRIs is between 18 and 30% [45] and is associated with a high risk of relapse [46]. Considering these conclusions, our findings only provide partial information regarding feasibility. There is a lack of rich qualitative data on long-term care, adherence and the adverse effects of pharmacological treatments. Findings from included studies were not able to discriminate experiences and acceptability of different types of antidepressants. Meanwhile, planned interruption has been discussed in depth but there is still a debate on the duration of pharmacological treatments [45].

The lack of clinical psychologists in primary may also impact the access and referral to recommended psychotherapies. In most western countries, psychotherapies are less used than medication regardless of preferences [47]. In our review only in 2 studies, both from Spain, [27, 28], the lack of access to psychotherapy was mentioned as a barrier to care by people with GAD. We have not included it as a finding as it was not triangulated in other contexts. Nevertheless, countries as Norway and the United Kingdom are starting programs to improve access to psychological treatments in PC by increasing the number of clinical psychologists [47] or training GPs in Internet-based-CBT [48]. Hopefully, these initiatives may impact on increasing feasibility of guideline implementation.

Our review provides information for efforts to improve the care of people with GAD in PC through interventions for patient empowerment and person-centred care. The elicitation of preferences for treatment options and the trade-offs they imply may improve adherence, as has been tested for other health conditions [49].

Issues related to the impact of treatment options on equity were also not explored in depth in the included studies. Our review found some specific evidence for elderly people with GAD, but an analysis of the acceptability of, preferences for and impact of treatment options by gender was not possible with the available data. Nevertheless, this QES only partially examined how social determinants of health affect the trajectories of people with GAD, taking into account that the prevalence of GAD is higher between the ages of 24 and 65, in women than in men (ratio 2:1), in people with lower incomes, in unemployed people, in women who perform domestic work and in people without a partner [1, 50]. Additionally, a meta-synthesis of qualitative evidence for mental health interventions in PC found inequalities in treatment access affecting vulnerable groups (e.g., migrants, asylum seekers, long-term unemployed people, homeless people, and elderly people with depression) [51].

The CERQual approach allowed us to improve the transparency of and confidence in the findings based on the decisions made in the review process. Findings with high and very high levels of confidence can support clinical practice guidelines (CPGs) and other evidence-based documents that use the GRADE framework to include aspects such as patient acceptability, values ​​and preferences, as well as implementation considerations [43]. Findings with low confidence levels may require additional supporting evidence.

Other aids that provide information to improve the empowerment of patients are CPGs. CPGs incorporate the values and preferences of the population and consider factors such as equity, acceptability and feasibility to determine the strength of recommendations based on the elaboration of patient decision aids (DAs) [47]. DAs are helpful for facilitating shared decision making in the shift towards a person-centered model and adapting population-based recommendations into individual patient-directed recommendations [52]. In addition, the development of DAs derived from CPG recommendations could offer a unique opportunity to improve clinical practice and increase patient satisfaction in the decision-making process related to their health and treatments [53].

### Limitations

The number of papers available (to us) was limited due to lack of funding and time constraints. Only Spanish and English papers could be reviewed due to a lack of funding for translation. This QES included studies published between 2008 and 2018, so we could only summarize the most recent scientific evidence to inform the treatment of people with GAD in PC, in which we identified qualitative research needs in addition to the abovementioned needs. The experiences and preferences of people with GAD with respect to recommended treatment options such as CBT, the stepped care model or combinations of pharmaceutical treatment and psychotherapy have been recently analysed sparsely or not at all.

Searching other databases or sources of grey literature search may also have found additional studies. To address the lack of available literature, indirect data were included in the review if they could inform the research question.

## Conclusions

Initial resistance to new treatments among people with GAD can make it difficult for them to access and adhere to treatment. Improving care may require patients to be informed of possible trajectories in stepped care pathways before the initiation of treatment so they are aware that they may need to try a number of options until the most effective treatment for them is found. Increased awareness of and information materials on GAD may facilitate both appropriate diagnosis and long-term care.

## Supplementary Information

**Additional file 1.**

## Data Availability

All data and materials can be accessed upon request to the corresponding author.

## Abbreviations

AD: Anxiety Disorder
ABP: Affect-focused Body Psychotherapy
CASPe: Critical Appraisal Skills Programme
CERQual: Confidence in the Evidence from Reviews of Qualitative Research
CBT: Cognitive Behavioural Therapy
CPG: Clinical Practice Guideline
CPGs: Clinical Practice Guidelines
DAs: Decision Aids
EFT: Emotion-Focused Therapy
GP: General Practitioner
GPs: General Practitioners
GAD: Generalized Anxiety Disorder
GRADE: Grading of Recommendations, Assessment, Development and Evaluation
PC: Primary Care
QES: Qualitative Evidence Synthesis
MI: Motivational Interview
SSRIs: Selective Serotonin Reuptake Inhibitors
SNRIs: Serotonin–Noradrenaline Reuptake Inhibitors
WHO: World Health Organization
