# Supplementary material for: Framing the process in the implementation of care for people with generalized anxiety disorder in primary care: a qualitative evidence synthesis
Source: BMC Fam Pract. 2020 Nov 20;21:237. doi: 10.1186/s12875-020-01307-6 (PMC7678131; doi:10.1186/s12875-020-01307-6)
Supplement: Supplementary file 1 — Additional file 1. [file 12875_2020_1307_MOESM1_ESM.doc]

**Table 2-5. Summary of findings**

Table 2: Theme 1: Trajectory of care

| **Summary of findings** | **Studies contributing to the findings** | **Methodological limitations** | **Relevance** | **Coherence** | **Adequacy** | **CERQual assessment** | **Explanation of CERQual assessment** |
| --- | --- | --- | --- | --- | --- | --- | --- |
| Finding a treatment or a combination of accessible, adequate and effective treatments required people with GAD and AD to try and to change several options over time. | Hurtado 2020,  Amor Mercado 2017,  HQO 2017, CPG GAD 2008 | Moderate concerns.  Two out of three studies had moderate limitations. One had minor limitations. | Moderate concerns.  Partially indirect findings for GAD, as the studies also included persons with depression and other ADs. | Minor concerns.  Data consistent within and across studies. | Moderate concerns.  One study offered sufficient data and two offered superficial data to this finding. | Moderate confidence. | Moderate concerns regarding methodological limitations and relevance.  Minor concerns regarding relevance and adequacy. |
| Starting any new treatment caused anxiety and resistance in the face of uncertainty. The degree of anxiety seemed to depend on previous knowledge and prejudices about the treatment. The resistance was usually resolved once the treatment was started. | O’Brien 2017, HQO 2017,  Cramer 2014,  Berg 2010 | Minor concerns.  Four studies had minor limitations. | Moderate concerns.  Partially indirect findings for GAD, as the studies also included persons with depression and other ADs. | Minor concerns  Data consistent within and across studies. | Minor concerns. Four studies offered sufficient data to this finding. | High confidence. | Minor concerns regarding methodological limitations, coherence and adequacy.  Moderate concerns regarding relevance. |

AD: *Anxiety Disorder*; GAD: *Generalized Anxiety Disorder*; GP: *General Practitioner;* CPG GAD: *Clinical Practice Guidelines for the Management of Patients with GAD in Primary Care*; HQO: *Health Quality Ontario*; PC: *Primary Care.*

**Table 3: Theme 2: Information needs**

| **Summary of findings** | **Studies contributing to the finding** | **Methodological limitations** | **Relevance** | **Coherence** | **Adequacy** | **CERQual assessment** | **Explanation of CERQual assessment** |
| --- | --- | --- | --- | --- | --- | --- | --- |
| People with GAD and AD did not receive enough information about their disorders, where to obtain help, what kind of help was available, what were the most effective treatments or how to handle the disease in everyday life and in times of crisis. | Amor Mercado 2017,  HQO 2014, Dickinson 2010,  CPG GAD 2008 | Moderate concerns. Two studies had moderate limitations. Two had minor limitations. | Moderate concerns.  Partially indirect findings for GAD, as the studies also included persons with depression and other ADs. | Minor concerns  Data consistent within and across studies. | Minor concerns. Four studies that together offered sufficient data to this finding. | High confidence. | Moderate concerns regarding methodological limitations and relevance.  Minor concerns regarding coherence and adequacy. |
| The delivery of information about the disease and care to people with AD, their relatives and their caregivers could be beneficial. However, some people with GAD did not always have or want this support (bearing in mind that the social or family context may be the source of the problem). | Amor Mercado 2017,  Bosman 2017, Cramer 2014, CPG GAD 2008 | Moderate concerns. Two studies had moderate limitations. Two had minor limitations. | Moderate concerns.  Partially indirect findings for GAD, as the studies also included persons with depression and other ADs. | Minor concerns.  The findings seemed to indicate the diversity and complexity of the phenomenon. | Minor concerns. Four studies offered sufficient data to this finding. | Moderate confidence. | Moderate concerns regarding methodological limitations and relevance.  Minor concerns regarding coherence and adequacy. |
| GPs expressed doubts about how to act during repeated anxiety crises and relapse. | Bosman 2016,  CPG GAD 2008,  Cook 2007 | Moderate concerns: One study had moderate limitations. Two had minor limitations. | Moderate concerns.  Indirect findings for GAD, as the studies only included persons with depression and ADs. | Moderate concerns. The interpretation of the finding was somewhat supported by data from two studies. No contradictory data were found in other studies nor in the literature review. | High concerns:  Three studies that together offered superficial data to this finding. | Low confidence. | Moderate concerns regarding methodological limitations, relevance and coherence.  High concerns regarding adequacy. |

AD: *Anxiety Disorder;* GAD: *Generalized Anxiety Disorder*; GP: *General Practitioner*; CPG GAD: *Clinical Practice Guidelines for the Management of Patients with GAD in Primary Care*; HQO: *Health Quality Ontario*; PC*: Primary Care*.

**Table 4: Theme 3: Preferences, acceptability and implementation considerations of treatment options**

| **Summary of findings** | **Studies contributing to the findings** | **Methodological limitations** | **Relevance** | **Coherence** | **Adequacy** | **CERQual assessment** | **Explanation of CERQual assessment** |
| --- | --- | --- | --- | --- | --- | --- | --- |
| **Preferences** | | | | | | | |
| There was variability in treatment preferences. Some people with GAD and AD preferred pharmacotherapy or a combination of pharmacotherapy and psychotherapy. Other people showed greater preferences for psychological treatments, but they differed in terms of the type of psychotherapy they preferred. They considered CBT, interpersonal therapy and support or group therapy to be more effective than other types of therapy. | Hurtado 2020,  Button 2019,  Amor  Mercado 2017,  HQO 2017,  CPG GAD 2008 | Moderate concerns. Two studies had moderate limitations. Two had minor limitations. | Moderate concerns.  Indirect findings for GAD, as the studies only included persons with depression and ADs. | Minor concerns.  Data consistent within and across studies. | Minor concerns.  Three studies that together offered sufficient data to this finding. | Moderate confidence. | Moderate concerns regarding methodological limitations, adequacy and relevance.  Minor concerns regarding and coherence |
| Treatment choice was based on the values and preferences of people with GAD since it increased the perception of effectiveness.  Preferences affected acceptability, uptake and adherence to pharmacological or psychological treatments. | HQO 2017,  Amor Mercado 2017,  Cramer 2014 | Moderate concerns.  One study had moderate limitations. Two had minor limitations. | Moderate concerns.  Partially indirect findings for GAD, as the studies also included persons with depression and other ADs. | Minor concerns.  Data consistent within and across studies. | Moderate concerns.  Two studies that together offered sufficient data and one that offered rich data. | Moderate confidence. | Moderate concerns regarding adequacy and relevance.  Minor or very minor concerns regarding methodological limitations and coherence. |
| **Acceptability of pharmacological treatments** | | | | | | | |
| Acceptability of antidepressant drugs depended on the perceptions of both patients and doctors regarding its effectiveness on symptoms relief. | Bosman 2016,  Dickinson 2010, Cook 2007 | Minor concerns.  Three studies had minor limitations. | Moderate concerns.  Indirect findings for GAD, as the studies only included persons with depression and ADs. | Moderate concerns.  Acceptability seemed to be variable between persons, conditions and moments and could have been based on other perceptions. | Minor concerns.  Three studies that together offered sufficient data to this finding. | Moderate confidence. | Moderate concerns regarding relevance and coherence.  Minor concerns regarding methodological limitations and adequacy. |
| The acceptability of and preference for drugs was based on patients’ and/or GPs’ beliefs that the health problem had physical/biological causes and therefore that the solution was medical rather than psychological. | Dickinson 2010 | Minor concerns.  One study had minor limitations. | Moderate concerns.  Indirect findings for GAD, as the studies only included persons with depression and ADs. | Minor concerns.  No contradictory data were found in other studies nor in the literature review. | Minor concerns. One study offered sufficient data to this finding. | Moderate confidence. | Moderate concerns regarding relevance and minor concerns regarding methodological limitations, coherence and adequacy. |
| Some people expressed few or no concerns when starting a pharmacological treatment, while others rejected drugs and refused to take them, as they were worried about their side effects and the stigma associated with their use. | HQO 2017,  Bosman 2016,  Dickinson 2010, Cook 2007 | Minor concerns.  Four studies had minor limitations. | Moderate concerns.  Partially indirect finding for GAD, as the studies also included persons with depression and other ADs. | Moderate concerns.  The findings did not seem to indicate the diversity and complexity of the phenomenon. | Minor concern. Three studies that together offered sufficient data to this finding. | Moderate confidence. | Moderate concerns regarding relevance and minor concerns regarding methodological limitations, coherence and adequacy. |
| Long-term acceptability was reduced in some people because they perceived that the drugs have limited capacity to eliminate GAD altogether. | Amor Mercado, 2017 | Moderate concerns.  One study had moderate limitations. | No or very minor concerns regarding relevance. | Minor concerns. Coherent with other findings within this review. | Moderate concerns. One study offered superficial data to this finding. | Moderate confidence. | No or very minor concerns regarding relevance and coherence moderate concerns regarding methodological limitations and adequacy. |
| GPs tended to recognize that social and emotional problems required non-pharmacological interventions that they could not always provide and expressed discomfort if they prescribed drugs in those situations. | Dickinson 2017 | Minor concerns.  One study had minor limitations. | Moderate concerns.  Indirect findings for GAD, as the studies only included persons with depression and ADs. | Minor concerns. No contradictory data were found in other studies nor in the literature review. | Minor concerns. One study offered sufficient data to this finding. | Moderate confidence. | Minor concerns regarding methodological limitations and coherence. Moderate concerns relevance and adequacy. |
| **Acceptability of psychological treatments** | | | | | | | |
| People with GAD had both positive and negative experiences with psychological therapies, which affected their acceptability. When previous experiences with the therapy were positive, their acceptability increased. | Amor Mercado 2017  HQO 2017,  Berg 2010 | Minor concerns.  Two studies had minor limitations. | Moderate concerns.  Partially indirect findings for GAD, as the studies also included persons with depression and other ADs. | Minor concerns. Coherent with other findings within this review. | Minor concerns. One study offered  sufficient data and another rich data to this finding | High confidence**.** | Minor concerns regarding methodological limitations and coherence.  Moderate concerns relevance and adequacy. |
| A positive relationship with the therapist facilitated acceptance and adherence to psychological treatment. The most accepted relationships with therapists were with clinical psychologists. With other professionals (physiotherapists), the relationships were more variable. | HQO 2017,  Marcus 2011,  Berg 2010 | Moderate concerns.  One study had moderate limitations. Two had minor limitations. | Moderate concerns.  Partially indirect findings for GAD, as the studies also included persons with depression and other ADs. | Major concerns. Experiences could be variable depending on the attending health professionals. | Minor concerns.  Three studies that together offered sufficient data to this finding. | Low confidence. | Minor concerns regarding methodological limitations and adequacy.  High concerns regarding coherence and relevance. |
| People with GAD considered motivational interviews to be acceptable. CBT or MI-CBT allows patients with GAD to assume a more active and engaged role in treatment. Offering tools and strategies to cope with to worry could condition the acceptability CBT or MI-CBT. | Button 2020  Marcus 2011 | Moderate concerns.  Two study had moderate limitations. | Moderate concerns.  One study was conducted in a private clinic by a specialized clinical psychologist. | Minor concerns. Coherent with other findings within this review. | Minor concerns. Two studies offered rich data to this finding. | Moderate confidence. | Moderate concerns regarding relevance and methodological limitations. Minor concerns regarding coherence and adequacy. |
| The acceptability of emotion-focused therapy depended on patients’ interpersonal difficulty expressing their emotions. | O’Brien 2017 | Minor concerns.  One study had minor limitations. | Moderate concerns.  The study was conducted in psychological services in PC that might not be available across contexts. | Moderate concerns. The finding was not supported by any additional literature. | Minor concerns. One study offered rich data to this finding. | Moderate confidence. | Moderate concerns regarding relevance and adequacy. Minor concerns regarding methodological limitations and coherence. |
| Experiences with body psychotherapy for people with GAD were variable. The feeling of uncertainty before a new treatment was increased, as body psychotherapy does not belong to a usual and well-known care path. | Berg 2010 | Minor concerns.  One study had minor limitations. | High concerns.  The study was conducted in psychiatric services by a physiotherapist and may not be transferable to PC. | Moderate concerns. The findings were not supported by any additional literature. | Minor concerns. One study offered rich data to this finding. | Low confidence. | Major concerns regarding relevance. Moderate concerns regarding adequacy.  Minor concerns regarding methodological limitations and coherence. |

AD: *Anxiety Disorder;* CBT: *Cognitive Behavioural Therapy*; GAD: *Generalized Anxiety Disorder*; GP: *General Practitioner*; CPG GAD: *Clinical Practice Guidelines for the Management of Patients with GAD in Primary Care*; HQO: *Health Quality Ontario*; PC*: Primary Care*.

**Table 5: Theme 4: Practices, acceptability and considerations of the interruption of treatment with antidepressants**

| **Summary of findings** | **Studies contributing to the findings** | **Methodological limitations** | **Relevance** | **Coherence** | **Adequacy** | **CERQual assessment** | **Explanation of CERQual assessment** |
| --- | --- | --- | --- | --- | --- | --- | --- |
| The use of antidepressants often lasted longer than recommended by reference guidelines. | Bosman 2016, Dickinson 2010,  Cook 2007. | Major concerns.  Qualitative studies are not the most appropriate design to address this finding. | High concerns.  Indirect findings for GAD, as the studies only included persons with depression and ADs. | Major concerns. The data were more varied than usual, and the description of the underlying problem was oversimplified. | Minor concerns  Three studies that together offered sufficient data to this finding. | Low confidence. | Major concerns regarding methodological limitations, relevance and coherence. Minor concerns regarding and adequacy. |
| Interruption of antidepressant treatment was not usually planned in PC. Practices related to the interruption of treatment were very variable and depended on the GP and his or her relationship with the person with AD. | Bosman 2016,  Dickinson 2010,  Cook 2007 | Minor concerns.  Three studies had minor limitations. | Moderate concerns.  Indirect findings for GAD, as the studies only included persons with depression and ADs. | Moderate concerns.  The findings did not seem to reflect the diversity of the phenomenon, as one PC centre planned an annual visit to review long-term antidepressant long term use. | Minor concerns. Three studies that together offered sufficient data to this finding. | Moderate confidence. | Minor concerns regarding methodological limitations and adequacy.  Moderate concerns regarding relevance and coherence. |
| Medication was sometimes passively continued due to inertia, routinization or the GP forgetting to review the medication. | Bosman 2016,  Dickinson 2010 | Minor concerns.  Two studies had minor limitations. | Moderate concerns.  Indirect findings for GAD, as the studies only included persons with depression and ADs. | Minor concerns.  No contradictory data were found in other studies or in the literature review. | Moderate concerns. Two studies that together offered slightly superficial data to the problem. | Moderate confidence. | Minor concerns regarding methodological limitations and coherence.  Moderate concerns regarding relevance and adequacy. |
| Perceptions of functional improvements such as a reduction in anxiety symptoms, improved sleep or increased stability were the basis of doctors’ and patients’ preferences to maintain drug treatment. Likewise, dependence and abuse of these drugs was a factor that was viewed negatively. | Bosman 2016, Dickinson 2010,  Cook 2007 | Minor concerns.  Three studies had minor limitations. | Moderate concerns.  Indirect findings for GAD, as the studies only included persons with depression and ADs. | Minor concerns.  The findings did not seem to indicate the complexity of the phenomenon. | Moderate concerns.  Three studies that together offered sufficient data to this finding. | Low confidence. | Moderate concerns regarding methodological limitations, relevance and adequacy.  Minor concerns regarding coherence. |
| The fear of relapse hindered the interruption of antidepressant treatment. | Bosman 2016,  Dickinson 2010, Cook 2007 | Minor concerns.  Three studies had minor limitations. | Moderate concerns.  Indirect findings for GAD, as the studies only included persons with depression and ADs. | Moderate concerns. The finding did not seem to indicate the complexity of the phenomenon. | High concerns  Three studies that together offered superficial data to the problem. | Low confidence. | Moderate concerns regarding methodological limitations and relevance.  Minor concerns regarding coherence and high concerns regarding adequacy. |
| Older people could have additional barriers to the interruption of pharmacological treatment, such as pessimism about the chronicity of the disease, negative expectations related to age or resistance to change. | Dickinson 2010 | Minor concerns.  One study had minor limitations. | Moderate concerns.  Indirect findings for GAD, as the studies only included persons with depression and ADs. | Moderate concerns.  The finding did not seem to indicate the complexity of the phenomenon. | Moderate concerns  One study offered slightly superficial data to the problem | Low confidence. | Minor concerns regarding methodological limitations.  Moderate concerns regarding relevance, coherence and adequacy. |
| Antidepressants were the most accessible treatment option, and GPs were reluctant to interrupt their use due to a lack of access to other therapeutic options. | Dickinson 2010 | Minor concerns.  One study had minor limitations. | Moderate concerns.  Indirect findings for GAD, as the studies only included persons with depression and ADs. | Coherent with other findings within this review. | Moderate concerns  One study offered slightly superficial data to the problem | Moderate confidence. | Minor concerns regarding methodological limitations and coherence.  Moderate concerns regarding relevance and adequacy. |
| GPs and people with GAD tried to identify a suitable time in terms of the patient’s stability and an appropriate reason to start the interruption. | Bosman 2016 | Minor concerns.  One study had minor limitations. | Moderate concerns.  Indirect findings for GAD, as the studies only included persons with depression and ADs. | Minor concerns.  The finding did not seem to indicate the diversity and complexity of the phenomenon. | Moderate concerns  One study offered slightly superficial data to the problem | Moderate confidence. | Minor concerns regarding methodological limitations and coherence  Moderate concerns regarding relevance and adequacy. |
| GPs and people with GAD needed information about antidepressant treatments, their recommended duration, and the reasons why treatment should be discontinued. | Bosman 2016, Dickinson 2010,  Cook 2007 | Minor concerns.  Three studies had minor limitations. | Moderate concerns.  Indirect findings for GAD, as the studies only included persons with depression and ADs. | Minor concerns.  The finding reflected the variety of data and was coherent with the information needs reported in other studies and literature reviews. | Minor concerns  Three studies that together offered sufficient data to this finding. | Moderate confidence | Minor concerns regarding methodological limitations, coherence and adequacy.  Moderate concerns regarding relevance. |
| An automated reminder or a specific appointment could help GPs initiate or manage the interruption of the antidepressant treatment. | Bosman 2016 | Minor concerns.  One study had minor limitations. | Moderate concerns.  Indirect findings for GAD, as the studies only included persons with depression and ADs. | Minor concerns. Coherent with additional literature. | Moderate concerns  One study offered slightly superficial data to the problem | Moderate confidence. | Minor concerns regarding methodological limitations and coherence.  Minor concerns regarding relevance. Major concerns regarding adequacy. |

AD: *Anxiety Disorder;* GAD: *Generalized Anxiety Disorder*; GP: *General Practitioner*; CPG GAD: *Clinical Practice Guidelines for the Management of Patients with GAD in Primary Care*; PC*: Primary Care*.
